# Rapid Mapping of Lithiation Dynamics in Transition Metal Oxide Particles with *Operando* X-ray Absorption Spectroscopy

**DOI:** 10.1038/srep21479

**Published:** 2016-02-24

**Authors:** Lea Nowack, Daniel Grolimund, Vallerie Samson, Federica Marone, Vanessa Wood

**Affiliations:** 1 Laboratory for Nanoelectronics, ETH Zürich, Zürich Switzerland; 2 microXAS, Paul Scherrer Institute, Villigen Switzerland; 3 TOMCAT, Paul Scherrer Institute, Villigen Switzerland

## Abstract

Since the commercialization of lithium ion batteries (LIBs), layered transition metal oxides (LiMO_2_, where M = Co, Mn, Ni, or mixtures thereof) have been materials of choice for LIB cathodes. During cycling, the transition metals change their oxidation states, an effect that can be tracked by detecting energy shifts in the X-ray absorption near edge structure (XANES) spectrum. X-ray absorption spectroscopy (XAS) can therefore be used to visualize and quantify lithiation kinetics in transition metal oxide cathodes; however, *in-situ* measurements are often constrained by temporal resolution and X-ray dose, necessitating compromises in the electrochemistry cycling conditions used or the materials examined. We report a combined approach to reduce measurement time and X-ray exposure for *operando* XAS studies of lithium ion batteries. A highly discretized energy resolution coupled with advanced post-processing enables rapid yet reliable identification of the oxidation state. A full-field microscopy setup provides sub-particle resolution over a large area of battery electrode, enabling the oxidation state within many transition metal oxide particles to be tracked simultaneously. Here, we apply this approach to gain insights into the lithiation kinetics of a commercial, mixed-metal oxide cathode material, nickel cobalt aluminium oxide (NCA), during (dis)charge and its degradation during overcharge.

In recent years, a variety of *in-situ* and *operando* techniques including atomic force microscopy^1^, Raman spectroscopy^2^,^3^,^4^, X-ray tomography^5^, X-ray absorption spectroscopy^6^,^7^,^8^,^9^,^10^ and X-ray diffraction^11^,^12^ have enabled, among other processes, visualization and quantification of lithium diffusion kinetics in lithium ion batteries (LIBs) during their operation^13^. Synchrotron-based X-ray absorption spectroscopy (XAS) shows particular promise for developing a deeper understanding of transition metal oxide compounds in LIBs^3^,^4^,^5^,^6^,^7^,^8^,^9^ because during lithiation and delithiation, the transition metals in the transition metal oxide active materials change their oxidation states. Most commercial LIB cathodes use layered transition metal oxide (LiMO_2_, where M = Co, Mn, Ni, or mixtures thereof) active materials that lithiate (or delithiate) via a process known as intercalation (or deintercalation), in which lithium atoms are inserted into (or extracted from) spaces in the crystal lattice. In contrast to materials that alloy with lithium, where the large changes in electron density and volume make it possible to study lithiation dynamics using absorption contrast (i.e. measurement of differences in absorption at a single energy) in 2D via transmission X-ray microscopy or in 3D via X-ray tomography, transition metal oxides that intercalate lithium exhibit a difference in absorption that is often too small for reliable tracking of the lithiation dynamics. However, the change in oxidation state that occurs during intercalation and deintercalation of lithium in these materials can be tracked by detecting shifts in the X-ray absorption near edge structure (XANES) spectrum of the transition metal. The potential of using XAS to study transition metal oxides in LIBs was recently highlighted, for example, by the work of Yang *et al.*, where the distribution of transition metals in commercial Li_1.2_Mn_0.525_Ni_0.175_Co_0.1_O_2_ was mapped before and after long-term cycling by XANES tomography, enabling the authors to obtain both structural and chemical insights into the degradation of this material^14^. Ideally, such studies could be performed *operando* and, indeed, the goal of our work here is to develop an approach that could facilitate *operando* XANES tomography.

Here we work in 2D and aim to demonstrate *operando* XAS measurements that simultaneously track lithium dynamics in a statistically significant number of particles with sub-particle spatial resolution. Such studies remain rare due to the challenge of balancing the need for fast (<5 min) XAS measurements that can yield insights into lithiation dynamics with the need for low dose measurements to prevent beam damage to the sample over the long investigation times required to track multiple charge and discharge cycles. As confirmed by recent studies of lithium iron phosphate with soft X-rays at the L-edge^15^ and explained in the Supplementary Information, the critical dose for the electrolyte and binder in LIBs, as for most organic materials, is 10^6^–10^8^ Gray^16^.

Traditionally, XAS measurements on LIBs have been performed with an unfocused beam. Such studies are below the critical dose and are fast, which enables *operando* measurements in transmission or fluorescence mode^17^,^18^, but yields an averaged XANES over the electrode such that no spatially resolved information can be obtained. Scanning a focused, micron-sized beam over the sample and measuring the fluorescence at multiple points provides spatial resolution at the subparticle-level, but results in a high local dose. Therefore, this approach is restricted to *ex situ* studies or a limited number of scans^7^. In addition, scanning a focused beam over the sample requires time^19^,^20^,^21^. While recent advances at beamlines have enabled quick extended x-ray absorption fine structure (QEXAFS) fluorescence measurements^22^,^23^ and time-resolved XANES in both transmission and fluorescence modes for biological and materials science applications^24^,^25^, these approaches would also require a high dose when implemented in scanning mode to achieve spatial resolution.

Full-field microscopy-based XAS provides an absorption map of the sample for every selected x-ray energy by taking a transmission image with a CCD camera after the partially attenuated X-rays for a specific energy are converted to visible light by a scintillator. This improves temporal resolution by eliminating the need for scanning and enables spatial resolution of a statistically significant number of particles^26^. To date, *in situ* XAS experiments on batteries in full-field setup have been performed with the intercalation compound LiFePO_4_^27^,^15^ and conversion materials NiO and FeF_3_^28^,^29^. To obtain sufficient absorption contrast for image processing, full-field XAS still requires a relatively high photon flux or long exposure time at every energy. While lower magnifications need less exposure time^30^, for a resolution of 20–40 nm, imaging times of 1–100 seconds per image are required.

In a typical measurement of a metal K-edge XANES spectrum, an energy resolution of at least 0.5 eV, which corresponds to about 50–100 different energies, is used in order to clearly visualize the absorption edge. Recording XANES spectrum at fewer energy points can significantly reduce the dose per scan. Although the features of absorption edge can no longer be readily identified by eye, this approach has been previously applied in XAS studies on NiO conversion reaction materials in LIBs^28^, NiO for fuel cells^31^,^32^, and oxidation of heated nickel powder^33^.

Here we demonstrate that combining full-field microscopy and a highly discretized energy resolution is a fast, low-dose approach to visualize the oxidation state of the particles contained in an electrode during electrochemical cycling. We use this approach to visualize the lithiation dynamics in commercial LiNi_0.8_Co_0.15_Al_0.05_O_2_ (NCA) at the particle- and electrode-level during (dis)charge as well as reduction of NCA and gas formation during overcharge. NCA is known for its high capacity of 180 mAh g^−1^ 34 compared to other transition metal oxide cathode materials including LiCoO_2_ (140 mAh g^−1^)^35^, LiMn_2_O_4_ (148 mAh g^−1^)^36^, LiFePO_4_ (170 mAh g^−1^)^37^. However, lithium ion diffusion in NCA is slower than in LiMn_2_O_4_, for example^36^,^38^. While this has been reported in computational^36^ and experimental electrochemistry and *in situ* XRD^39^ studies, the local influence of slow lithium diffusion kinetics has never been visualized at the particle level. Likewise, while it is known that transition metal oxide materials are subject to oxygen loss during overcharge, which results in decreased battery performance, fast ageing and, in worst case, thermal runaway^40^,^41^, this has never been directly visualized.

## Results and Discussion

### Measurement Approach and Validation

**Figure 1:**
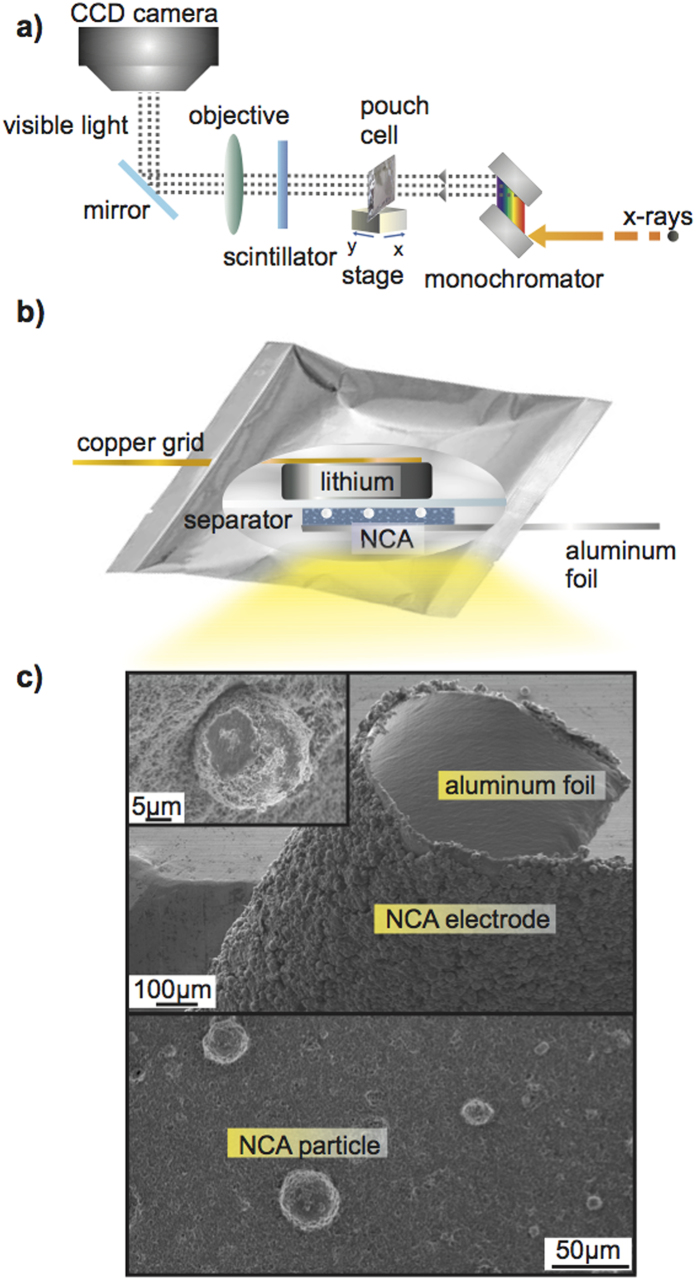
The experiment setup. Schematics of (**a**) the beamline configuration and (**b**) the pouch cell contents showing the copper current collector mesh on lithium metal, the separator soaked with LP30 electrolyte, and the NCA monolayer electrode on an aluminum foil. (**c**) Scanning electron microscopy (SEM) images of the NCA electrode on the aluminum foil current collector coated with the electrode of PVDF binder, carbon black, and NCA layer, including a close-up of single particles (left corner) and the top surface of the electrode showing well-separated NCA particles (bottom image).

Our set-up (Fig. 1a) is similar to that of other full-field XAS measurements. As explained in the Methods, measurements are performed in transmission mode using an unfocused beam that illuminates a 1500 × 1500 micrometer sample area. At each voltage of interest, we record the transmission images through a pouch cell (Fig. 1b) as a function of energy covering the XANES region of the element of interest^42^. For each pixel, we obtain an absorption spectrum *μ*(*E*) calculated using 
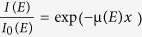
, where *I*_*0*_(*E*) and *I*(*E*) are the incident and attenuated X-ray intensities, respectively. The effect of different active material thickness, *x*, across the electrode is removed by normalizing the absorption edge from pre- to the post-edge region to 1. Typically, XAS measurements are done with high energy-resolution such that the oxidation state can be directly determined by tracking the shift in the XANES. However, such high-energy resolution spectra take time (often prohibitively long for an *operando* measurement where a cell is cycled faster than 1C (i.e., 1 hour for (dis)charge), necessitating measurements well below 5 minutes, and expose the cell to high doses. The approach we demonstrate is to identify energies around the XANES with key features, take measurements at these energies, and obtain a discrete absorption spectrum *μ(E*_*1*_*,…,E*_*n*_) for each pixel. During post-processing, the discrete absorption spectra *μ(E*_*1*_*,…,E*_*n*_) are compared to high energy-resolution XANES of charged and discharged references to identify the extent of (dis)charge across the imaged area at every voltage step.

To demonstrate and validate this rapid approach, we investigate commercial NCA microspheres with a particle size distribution mode at 20 μm +/− 7.7 μm. Detailed physical and electrochemical characterization of these particles is provided in Supplementary Figure S1. In NCA, nickel undergoes oxidation from Ni^3+^ to Ni^4+^ during delithiation (charge) in the range of 2.8–4.3 V^43^. As cobalt remains Co^3+^ in this voltage range, and aluminum serves as a dopant to stabilize the crystal lattice, we only need to scan energies near the nickel edge around 8.33 keV. As shown in Fig. 1c the electrode is fabricated with 50 wt% NCA particles dispersed in a monolayer, which facilitates single particle identification in 2D. The cell is sealed in a multilayer composite foil, which serves as a barrier to oxygen and water. Because this foil is transparent at 8.33 keV, no kapton window in the cell is required, which improves electrochemical stability.

**Figure 2:**
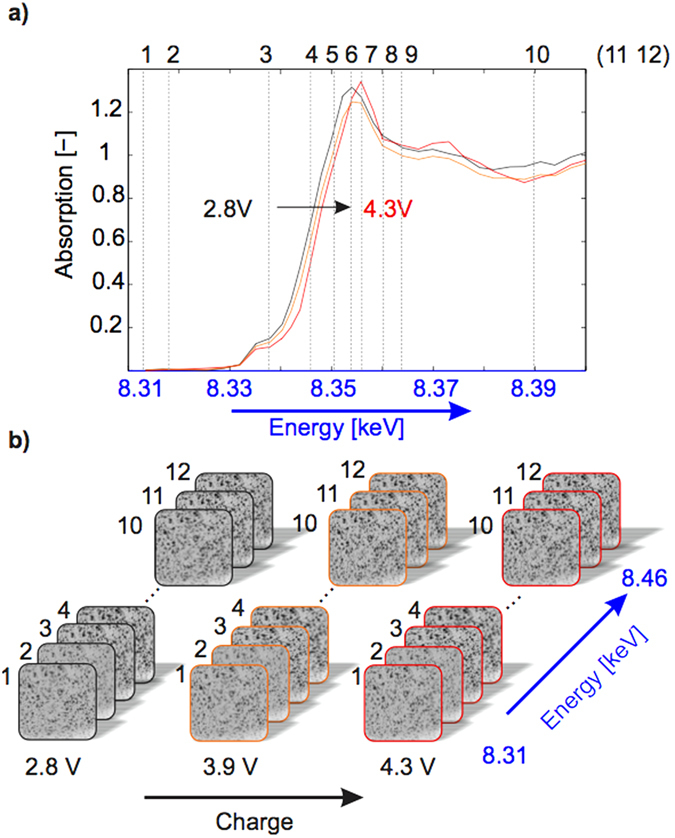
Schematic of the XAS measurement approach. (**a**) High resolution absorption spectra obtained from a charged cell at 4.3 V (red) and a discharged cell at 2.8 V (black). The orange curve is from a sample at 3.9 V (SOC of 0.6). Dashed lines indicate 10 of the 12 energies used for the fast scan. The two additional scans are performed at higher energies (8.447 and 8.460 keV) and used to normalize the absorption spectra. (**b**) Three sets of transmission images taken at selected energies for different SOCs (0, 0.6, and 1). The particles become “darker” at higher energies (i.e. above the edge), where absorption is greater.

We first obtain reference absorption spectra between 8.27 keV and 8.6 keV with high energy resolution (energy steps of 0.5 eV around the edge and 2 eV above the edge) for a fully charged (4.3 V) and discharged (2.8 V) cell held at the designated voltage by a potentiostatic step. These spectra contain information averaged across the illuminated cell area. A slow C/24 charge ensures enough time for lithium diffusion to take place such that the full capacity is reached (Supplementary Figure S5). These reference spectra are collected in fluorescence mode for optimal signal. As expected, these spectra show an absorption edge offset of several eV, which reflects the shift from NCA with primarily Ni^3+^ in a discharged cell to NCA with primarily Ni^4+^ in a charged cell. In our approach, we select 12 energies (indicated in Fig. 2a) and collect transmission images at these energies (Fig. 2b) to obtain discrete absorption values *μ(E*_*1*_*,…,E*_*12*_) for each pixel. The two energy points below and the two above the edge are needed for accurate normalization of the spectrum. The eight points in the near edge region are selected as the points with the greatest difference between the Ni^3+^ and Ni^4+^ reference spectra. The number of energy points near the edge could be reduced from eight points; however, we found that eight points enables us to reliably account for any background fluctuations coming from changes that occur in the electrochemical cell during operation (e.g. metallic lithium thickness in the pouch cell changes during electrochemical cycling). As described in the Methods, for each pixel, we then fit the normalized *μ(E*_*1*_*,…,E*_*12*_) to a linear combination of the charged (*μ*_*4+*_(*E*)) and discharged (*μ*_*3+*_(*E*)) reference measurements:


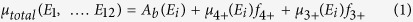


The local background attenuation *A*_*b*_(*E*_*i*_) accounts for small differences in the amount of the polymer binder, carbon black, and electrolyte, at each location in the electrode due to the spherical shape of the particles. *A*_*b*_(*E*) is at maximum 2% and, on average, less than 0.1%. Plotting *f*_4+_ or *f*_3+_ for each pixel gives an oxidation state map showing the spatial location of Ni^4+^ or Ni^3+^ in the electrode. Furthermore, each image constitutes a dose of only ~33 Gray for the organic carbonate electrolyte and polymer binder, which means that >1000 images can be taken before exceeding the critical dose. This corresponds to 100 SOC measurements throughout an electrochemical cycling profile.

**Figure 3:**
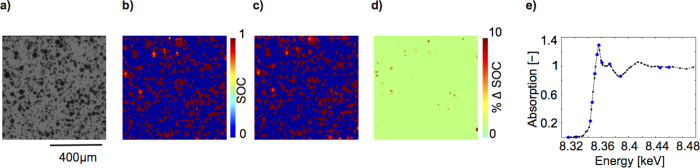
Validation of the accuracy of the 12-point scan approach. (**a**) Transmission image at E = 8.36 keV, showing sample area considered of a completely charged cell. (**b**) State of charge (SOC) map obtained from high resolution scan with 47 energy steps. (**c**) SOC map of the same sample area obtained using only 12 energy steps. (**d**) Difference in SOC determined using the two different types of scans, showing an average difference over the entire scan area of below 1%. (**e**) The obtained spectra averaged over all particles in the scan area from the 47 step (black) and the 12 step measurement (blue circles).

To demonstrate the accuracy of this rapid approach, we compare results from our 12-energy point measurement to those of a high energy resolution scan with 47 points on the same area of electrode (Fig. 3a) held at the same SOC. The SOC map from the high energy resolution measurement (Fig. 3b) is comparable to that of the low resolution measurement (Fig. 3c), deviating on average by less than 1%. (Fig. 3d). The absorption spectra from both measurements averaged over all pixels corresponding to particles are compared in Fig. 3e.

### Visualization of Lithiation Kinetics in Nickel Cobalt Aluminum Oxide-based Cathodes

**Figure 4:**
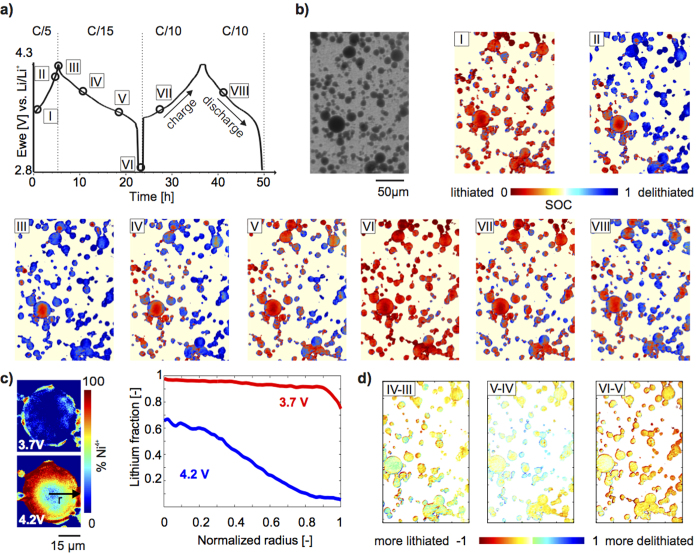
State of charge (SOC) mapping during electrochemical cycling. (**a**) Electrochemical cycling profile. (**b**) Transmission image of a region of electrode for which SOC maps for points I–VIII during the electrochemical cycle shown in panel (**a**). False colour indicates extent of lithiation (red) or delithiation (blue). (**c**) Left: Ni^4+^ oxidation maps of a single large particle during charge at 3.7 V and 4.2 V (times steps I and II in panel (**a**). A ring-like delithiation pattern is already visible at 3.7 V. Right: Radially integrated fraction of lithium in the particle at 3.7 V and 4.2 V as a function of distance from the center of the particle (r = 0) showing inhomogeneous lithiation in large particles at C/5. (**d**) Map of the difference in lithium content between the sequential timesteps III and IV (left), IV and V (middle), and V and VI (right). The overall lithium concentration is increasing, but is locally redistributed.

Having demonstrated that the 12 energy points chosen are representative for the XANES, we perform these 12 energy-point measurements while charging and discharging an NCA electrode according to the profile shown in Fig. 4a, and, in Fig. 4b, plot the spatial map of the local SOC at the different time steps labelled as time steps I–VIII in Fig. 4a. While we observe almost complete lithiation across the sample after discharge (time step VI), after charging to 4.3 V (time step III), the centre portion of large particles remains lithiated, which is consistent with electrochemical cycling data where charging to 4.3 V at C/5 gives about 90 % of the theoretical capacity (Figure S5). Figure 4c presents close up oxidation maps of a large particle, showing the amount and spatial location of Ni^4+^ (delithiated NCA) at 3.7 V (time step I) and 4.2 V (time step II). Plotting the amount of lithium left in the particle as a function of distance from the particle centre (r = 0) shows that the ring-like delithiation profile is present throughout the charging step. This is indicative of slow lithium diffusion kinetics during first charging (delithiation), which is in agreement with recent experimental findings by in *operando* XRD studies averaged over a NCA electrode^39^. Figure 4d plots the difference in lithiation between sequential time steps III and IV (left), IV and V (middle), and V and VI (right) during discharge. As expected, the overall lithium concentration increases at each time step, seen by the fact that on average the shading of each map is reddish (indicating a decrease in the SOC between time steps). This data visualization approach also enables us to observe how the lithium redistributes locally. In particular, we find that while lithium predominately moves from edge into the middle of the particle, during the middle of the discharge cycle, lithium leaves the edge of small particles and relocates to large particles. Therefore this method can track inhomgenieties in lithiation.

### Visualization of Degradation during Overcharge

**Figure 5:**
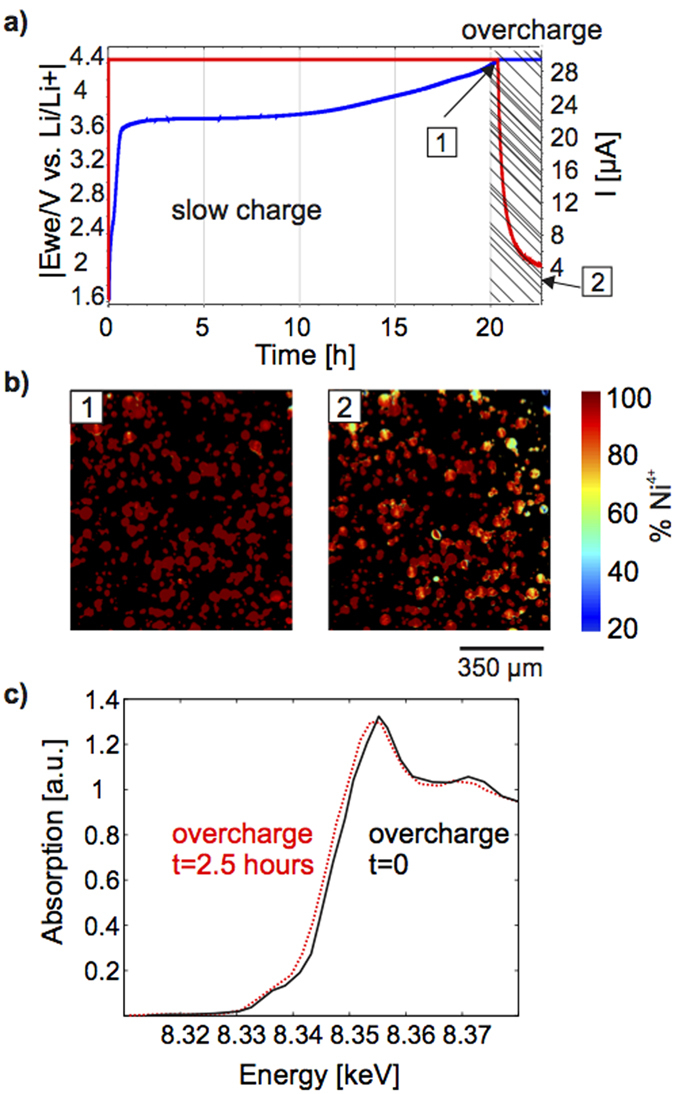
NCA during overcharge. (**a**) The charging profile to 4.4 V and the potentiostatic step for 2.5 hours at 4.4 V (blue) and the corresponding cell current (red). The shaded region indicates the time during with the particle is in overcharge, point 1 marking the beginning of overcharge and point 2 marking 2.5 hours of overcharge. (**b**) Maps of the oxidation state showing that particles charged to 4.4 V are completely delithiated (left) and the same particles after 2.5 hours at 4.4 V are partially reduced (right). (**c**) XANES averaged over all particles in a 0.56 mm^2^ subarea of the electrode before (black) and after 2.5 hours (red dashed) of overcharge showing an edge shift to lower energies, indicating that reduction occurs during overcharge.

Since overcharge of batteries poses a major reliability and safety concern for battery manufacturers and end applications developers^43^,^44^,^45^, we also investigated our NCA particles under “soft” overcharge conditions (4.4 V). NCA is usually only used up to 4.3 V, since use at higher voltages results in reduction of the Ni^4+^ to Ni^3+^ and release of oxygen, which reacts further with the electrolyte to form carbon dioxide causing drying out of the electrode or unwanted swelling of the cell^41^,^46^,^47^,^48^. While overcharge has been studied by differential scanning calorimetry (DSC)^46^ and X-ray diffraction (XRD)^41^ of overcharged cathodes and by gas chromatotography^41^ of the electrolyte, we investigate whether particle reduction and gas formation^39^,^49^ can be observed and spatially localized with XAS. We map the oxidation state of a cell after 1 minute and again after 2.5 hours of overcharge at 4.4 V (Fig. 5a). To ensure that the effects observed come from overcharge conditions and that there is no acceleration of reduction or gas evolution from prolonged radiation exposure, the sample is moved out of the beam while being held in overcharge. After 2.5 hours and 15 mAh g^−1^ overcharge it is evident that some particles are reduced (higher Ni^3+^ content) (Fig. 5b). The calculated change in the amount of Ni^3+^ over the imaged area is 5.7 %, which corresponds to 10.2 mAh g^−1^ in the reduction of the NCA after complete delithiation. Furthermore, knowing that our electrode contains 2.202 mg NCA, we can use Faraday’s law to approximate the mass *m* of oxygen released during this overcharge using: 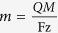, where *Q* is the charge put in the overcharge (81 mC), *M* is the molar weight of oxygen (32 g mol^−1^), and *z* is the number of electrons (4). We expect about 10 μg of oxygen, which would occupy a volume of about 7 μl. This volume corresponds to about 5 % of the amount of electrolyte injected into the cell. Indeed, gas formation is detected in the transmission images (See Supplementary Video). Therefore we believe the reduction of the nickel could be explained by overcharge reactions and could be used as a tool to study even more complex reactions during abuse.

In summary, we demonstrate the reliable and rapid characterization of SOC of intercalation compounds in LIBs during electrochemical cycling using full field transmission X-ray microscopy at only 12 energies. Using NCA as an example material, we show that this technique can shed light on the lithium kinetics, resolving the change in oxidation state during overcharge of NCA particles and the corresponding oxygen release. Such measurements offer the low radiation doses and fast measurement times needed to image processes in batteries *operando*. While spherical particles were used here to assist with particle identification, this technique is not limited by particle shape or size, and the resolution is set by the beamline optics. Furthermore, this approach can be applied to study mixed transition metal oxide materials (e.g LiNi_0.33_Mn_0.33_Co_0.33_O_2_ (NMC)) or mixed cathodes containing two transition metal oxide materials (e.g. NCA and LiMn_2_O_4_ (LMO)) by performing discretized energy scans near the cobalt edge (7.71 keV) and/or manganese edge (6.54 keV).

## Methods

### Experimental Setup & Measurements

Experiments are performed at the microXAS (X05LA) beamline of the Swiss Light Source at the Paul Scherrer Institute. The incoming photon flux at the beamline is about 3 × 10^12 ^ph s^−1^ at the sample after passing through the double crystal silicon monochromator system (Kohzu Co.), which provides an energy resolution ΔE/E of 0.02%. After passing through the pouch cell, the attenuated X-rays hit a YAG:Ce-doped scintillator, where the X-ray photons are converted to visible light, which is magnified 10-fold and imaged by a CCD (pco. 2000, 2048 × 2048 pixel, 7.4 um pixel size, 6 e^−^ rms at 10 MHz). This configuration provides a field of view of ca. 1500 × 1500 micrometer, with one pixel corresponding to approximately 0.74 × 0.74 μm^2^. For calibration and reference measurements on the nickel standards (nickel foil, NiO, LiNiO_2_, and NiO_2_) and the fully charged and discharged NCA electrodes high resolution XANES scans are taken from 8.31 keV to 8.5 keV in 0.5 eV steps near the edge and in 2 eV steps above the edge in fluorescence mode (silicon drift diode (SDD) detector, single element (Ketek GmbH, Germany)) and are processed in Athena IFEFFIT^50^ (Supplementary Figure S4).

For the *operando* measurements, images are taken at 12 energies: 8.31 and 8.322 keV in the pre-edge region; 8.343, 8.346, 8.350, 8.353, 8.356, 8.360, 8.372, and 8.388 keV in the near edge region; and 8.447 and 8.460 keV for calibration above the edge. At each energy, a total of 20 images with each 150 ms exposure time are taken: 10 of the sample followed by 10 with the sample moved out of the beam (flats). This results in a sample exposure time of 1.5 s and, with the time to move the sample out of the beam, a total time of 50 s at each energy. Therefore the measurement is currently limited by the time for the sample movement and deadtime of the camera. Measurement time could be improved in future experiments to less than a minute. Image processing is done with Fiji ImageJ. The ten sample images are averaged and divided by the averaged flats to account for fluctuations in the beam. The averaged images are aligned by a scale invariant feature transform (SIFT) algorithm to correct any shift caused by the movement of the sample, the x-y stage, or the X-ray source and optics. To validate this approach, the 12-point method is compared to fine scans with 47 images taken with 0.5 eV step size and 2 eV step size above the edge from 8.3 to 8.46 keV.

### Data Analysis

Full-field transmission images are processed in MATLAB. A particle-free area is chosen to calculate the attenuation of the non-NCA components of pouch cell: aluminum foil, binder, carbon black, electrolyte, PE separator, multilayer foil, and lithium (Supplementary Figure S6). The aluminum foil for example, contains traces of iron, which absorbs in the energy window of interest and needs to be corrected. This background attenuation is then subtracted from all *μ(E*_*i*_). To associate a pixel with a particle, we set the criterion that the absorption difference over the edge must be four times larger than the background noise.

The absorption spectrum for each pixel is then normalized by subtracting a linear fit of the pre-edge region (8.310 & 8.322 keV) and the post-edge region (8.447 & 8.460 keV) and by defining the difference between the pre-edge and post-edge to 1. This removes the effect of different active material thicknesses across the electrode. The good correspondence between the gray scale transmission images in which the particles are dark and the SOC maps, showing particles in false colour, confirms the validity of the approach (Fig. 4b).

The discrete spectra are fit to the references according to equation 1. For each pixel, different combinations of the reference Ni^3+^, Ni^4+^, and background contributions are considered in 2% steps 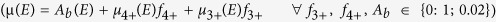. The best fit out of the 1326 possible combinations is determined by selecting the combination of values representing the ratio of the reference states with the minimal difference to normalized absorption data *μ(E*_*1*_*…E*_*i*_) for all energies, indexed by *i*, where *i* = 12 or 47 depending on the scan.


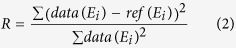


We require an R-value below 0.02 to accept a combination of curves as a fit. A representative plot of the fitting value *R* is given in Supplementary Figure S7. Three types of maps convey information about the location of lithium within the sample. Oxidation state maps (e.g. Figs 4c and 5b) gives the percent of Ni^4+^ (100 × *f*_4+_) in the slice of material imaged in a given pixel. SOC maps (e.g. Figs 3b and 4b) show the fraction of lithiated and delithiated material imaged in a given pixel by plotting


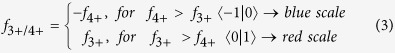


These can then linearly be plotted on a scale from 0 to 1 to show the local SOC. Subtracted maps (e.g. Fig. 4d) show the difference in SOC between two time steps and have values from −1 (less lithiated) to 1 (more lithiated).

## Additional Information

**How to cite this article**: Nowack, L. *et al.* Rapid Mapping of Lithiation Dynamics in Transition Metal Oxide Particles with *Operando* X-ray Absorption Spectroscopy. *Sci. Rep.* **6**, 21479; doi: 10.1038/srep21479 (2016).

## Supplementary Material

Supplementary Information

Supplementary Video
