# Supplementary material for: Rapid Mapping of Lithiation Dynamics in Transition Metal Oxide Particles with Operando X-ray Absorption Spectroscopy
Source: Sci Rep. 2016 Feb 24;6:21479. doi: 10.1038/srep21479 (PMC4764840; doi:10.1038/srep21479)
Supplement: Supplementary Information [file srep21479-s1.pdf]

Supplementary Information for

Rapid Mapping of Lithiation Dynamics in Transition Metal Oxide Particles with Operando X-Ray Absorption Spectroscopy

Authors and Affiliations:
Lea Nowack[1], Daniel Grolimund[2], Vallerie Samson[2], Federica Marone[3], Vanessa Wood[1]

[1] Laboratory for Nanoelectronics, ETH Zürich, Zürich Switzerland
[2] microXAS, Paul Scherrer Institute, Villigen Switzerland
[3] TOMCAT, Paul Scherrer Institute, Villigen Switzerland

Contents

Section A. Electrode Preparation

Section B. Calculating X-ray Dosage

Figure S1 Degradation in a Focused Beam

Section C. NCA Characterization

Figure S2 SEM and Particle Size Distribution
Figure S3 Electrochemical cycling

Section D. XAS Experiment

Figure S4 Sample holder and beamline setup
Figure S5 Oxidation state references
Figure S6 Electrochemistry in pouch cell during XAS
Figure S7 Background calibration
Figure S8 Quality of fit

Supplementary Video

A. Electrode Preparation

Electrodes are fabricated by doctor-blading a slurry with 50wt% NCA, 20wt% carbon black, and 30wt% PVDF to a wet thickness of 100 μm on a 10-μm aluminum foil. The slurry is dried overnight under vacuum at 80 °C.

B. Calculating X-ray Dosage

The dose rate *D* is dependent on the flux and energy absorbed[15]:

$$D = \frac{N}{A} * \frac{\mu E}{\rho}$$

where *N* is the number of incident photons per second (here 3×10^{12} ph s^{-1}), *A* is the examined area (here 0.5625 mm^2, μ is the linear attenuation coefficient taken from NIST (here, 6.56 cm^{-1} for DMC and 9.18 cm^{-1} for PVDF), ρ is the density of the material (here, 1.78g cm^{-3} for PVDF and 1 g cm^{-3} LP30 electrolyte), and *E* is the energy. For an energy of 8.46 keV, this gives about 560 Gray for PVDF and 710 Gray for DMC per image. The absorbed dose for 20 μm PVDF is 18 Gray and for 70 μm of DMC 33 Gray. In comparison, reducing the beam to 1μm^2 results in a local absorbed dose in the range of 10^7 Gray, which means only one scan is possible.

Figure S1 shows an image of a particle taken during exposure to a focused beam. We note that this degradation does not immediately show up on most electrochemical measurements because the degradation due to a focused beam occurs only in a very small volume (~50 μm^3) and electrochemical performance is measured over the entire electrode (~50 μm x 2 cm x 2 cm). Degrading the ~1/1000000000th of the electrode volume will not effect the overall electrochemistry, but it will effect the electrochemistry locally at the particular particle being imaged. This means that what is being visualized is not representative of the overall behavior of the electrode.

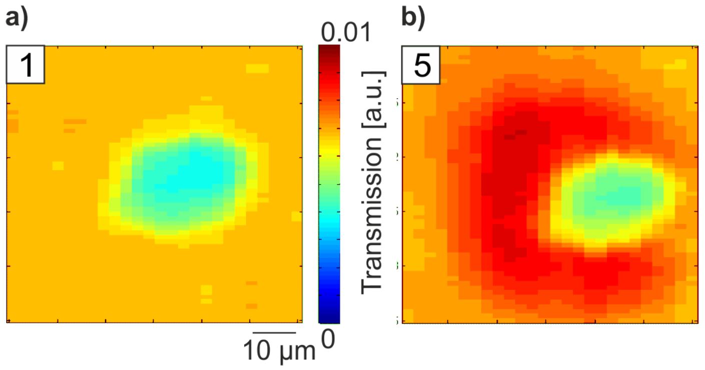

Figure S1 Images of a battery particle taken by a focused beam with 1 µm beam size. After 5 scans beam damage is clearly visible in transmission (not in fluorescence) in the system. Slight differences already develop during the 3rd scan. We therefore assume that the system is influenced by this measurement technique after 2 scans and observed this for several samples.

C. NCA Characterization

The NCA particles are obtained from an industrial partner and contain $LiNi_{0.8}Co_{0.15}Al_{0.05}O_2$. Scanning electron microscopy (SEM) images are taken using a Hitachi S4800 and SEM-focused ion beam (FIB) tomography is performed with FEI Company Helios NanoLab 450s. The particle size analysis is done using a Sympatec Helios particle size analyzer based on volume frequency distribution. Electrochemical characterization is carried out on electrodes of the same batch as the ones used in the experiments at the beamline.

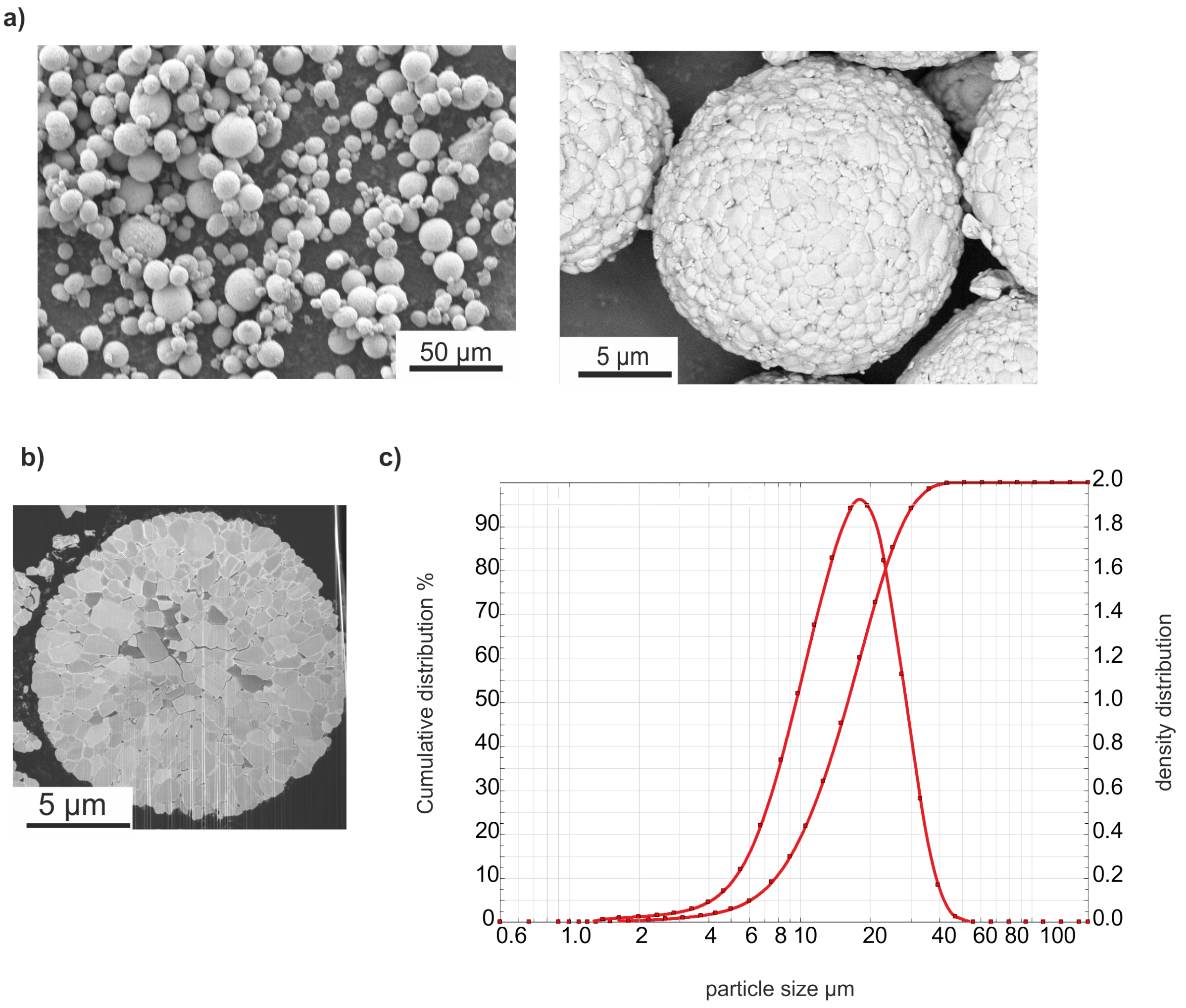

Figure S2 Characterization of NCA particles a) SEM images of NCA particles. b) FIB crossection through a NCA particle c) Particle size distribution of NCA particles based on volumetric frequency distribution.

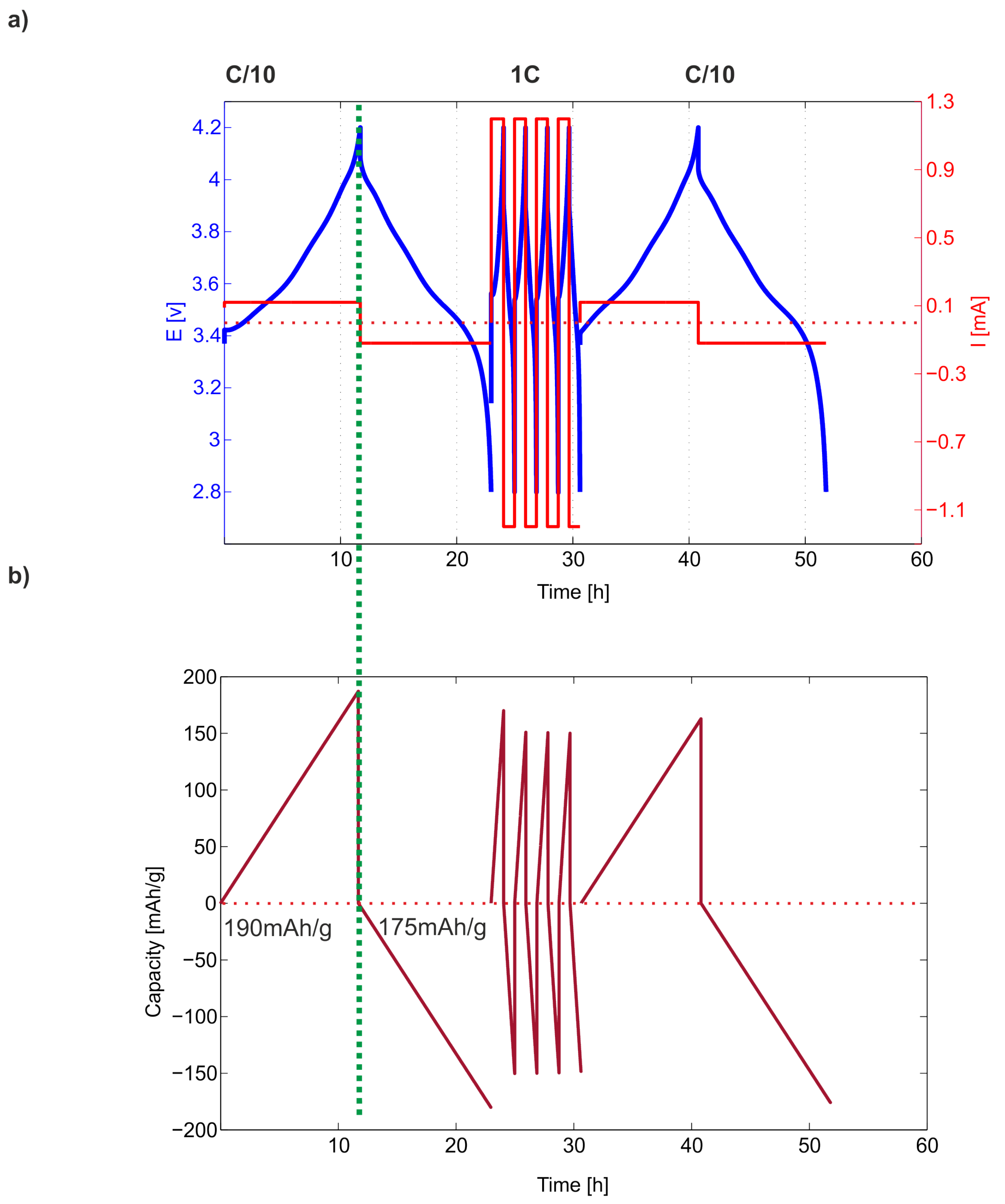

Figure S3 Electrochemical cycling of NCA versus lithium in a stainless steel cell in the laboratory. a) Voltage, current, and b) capacity during C/12 and 1C cycling. At 1C, 84% of the maximum capacity is still reached. The capacity after the first charge shows higher capacity (190 mAh g^{-1}) than during subsequent charge cycles (175 mAh g^{-1}) consistent with Ref. 19.

Section D. XAS Experiment

This section provides details of the XAS experimental setup and data processing.

Electrodes with a 1.3-cm^2 punched hole are fixed to a 4-mm Al tap (MTI Corp) with electrolyte-resistant tape (MTI Corp.). In an argon glovebox, the electrode and a PE separator (Celgard K1650) are soaked with electrolyte (1M $LiPF_6$ in 1:1 EC:DMC) and assembled in a pouch cell with a Li counter electrode pressed on a copper mesh. The pouch cell material is a PETmet/PE/PETmet/LDPE/EVOH (12/15/12/75)-µm multilayer foil (Gruber Folien). The PETmet consists of PET covered with a thin (less than 10 nm) Al layer as an oxygen and water barrier[27]. To maintain pressure in the cell, and preserve electrochemical cycling stability, this pouch cell is placed in a holder **(Figure S4)**, which sandwiches the pouch cell between a rubber plate and a wave spring, maintaining the inside pressure of the cell necessary for stable electrochemistry. A 1.3 mm-diameter hole in the holder allows beam transmission. The cells are cycled with a Biologic SP150.

A photograph and schematic of the beam line is also shown in **Figure S4**. The horizontally deflecting collimating torroidal mirror is placed ~18m from the source. Cryo-cooled, vertically deflecting, fixed-exit double crystal monochromator equipped with Si(111) crystals is located at ~24 m from the source. Two two-dimensional collimation guard slit systems are located at 31 and 37 m respectively (or 1 and 7m upstream the sample). The scintillator is located 0.03 m downstream the sample plane.

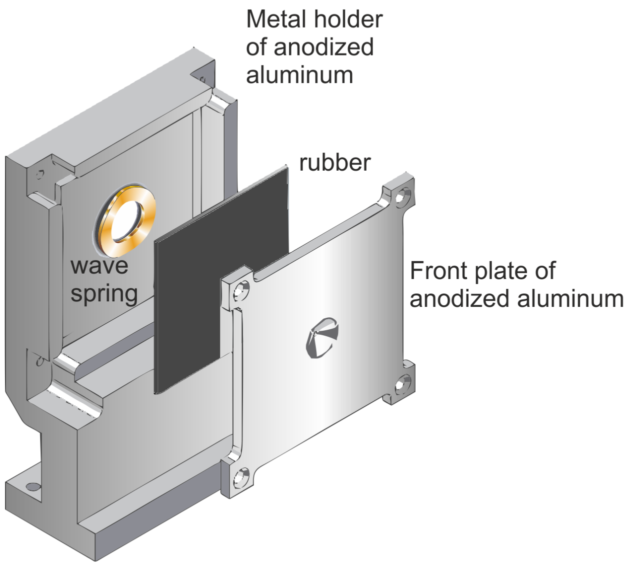
Metal holder of anodized aluminum
rubber
wave spring
Front plate of anodized aluminum

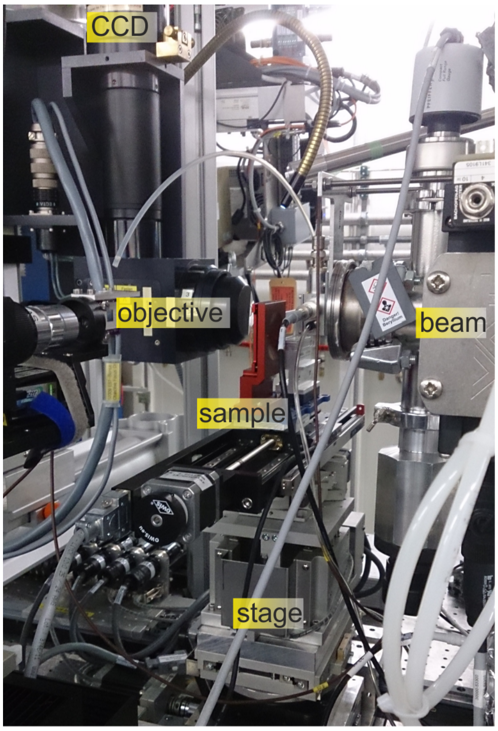
CCD
objective
beam
sample
stage

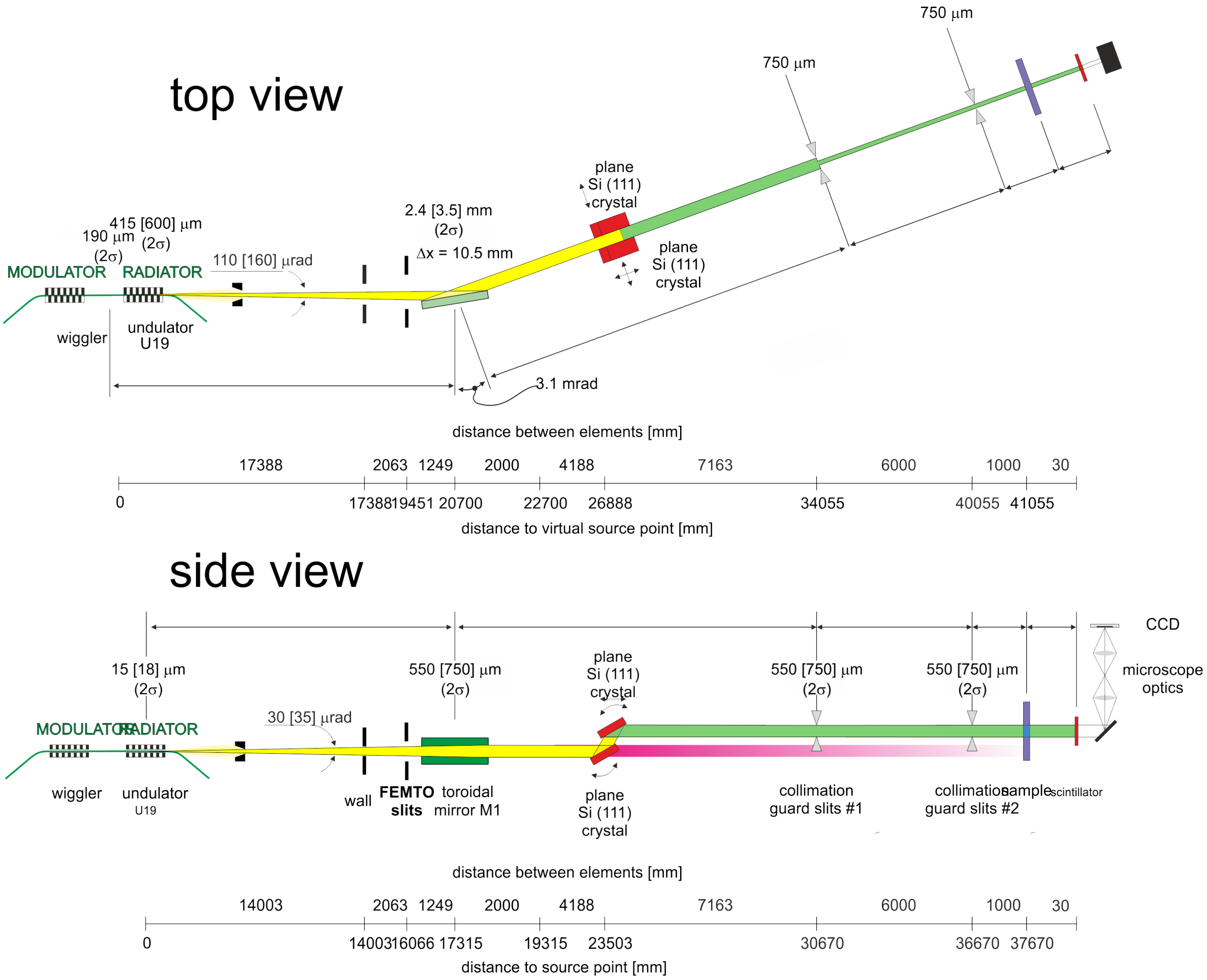
top view
750 μm
750 μm
plane Si (111) crystal
plane Si (111) crystal
415 [600] μm (2σ)
190 μm (2σ)
2.4 [3.5] mm (2σ)
Δx = 10.5 mm
110 [160] μrad
MODULATOR
RADIATOR
wiggler
undulator U19
3.1 mrad
distance between elements [mm]
17388
2063
1249
2000
4188
7163
6000
1000
30
0
17388
19451
20700
22700
26888
34055
40055
41055
distance to virtual source point [mm]
side view
15 [18] μm (2σ)
550 [750] μm (2σ)
plane Si (111) crystal
550 [750] μm (2σ)
550 [750] μm (2σ)
CCD
microscope optics
30 [35] μrad
MODULATOR
RADIATOR
wiggler
undulator U19
wall
FEMTO slits
toroidal mirror M1
plane Si (111) crystal
collimation guard slits #1
collimation guard slits #2
sample
scintillator
distance between elements [mm]
14003
2063
1249
2000
4188
7163
6000
1000
30
0
14003
16066
17315
19315
23503
30670
36670
37670
distance to source point [mm]

Figure S4 Sample and beamline setup. Left: Sample holder for pouch cell, which is placed between the spring and the rubber plate to guarantee a uniform pressure distribution. All metallic parts are anodized to avoid short-circuiting of the sample. Right: Photograph of the beamline setup (anodized sample holder is red). Top and side-view schematics of the beamline.

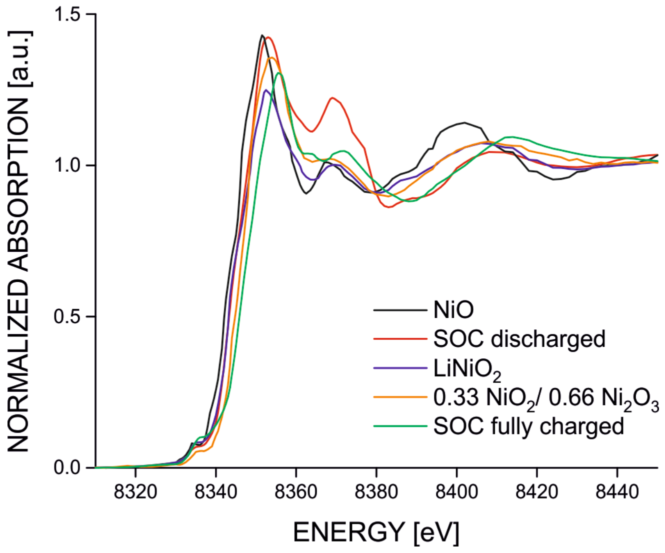

Figure S5 XANES of nickel references. Ni(IV)O_2, LiNi(III)O_2 and Ni(II)O powders (from Sigma Aldrich) are compared to the XANES of charged (primarily Ni^{4+}) and discharged (primarily Ni^{3+}) NCA particles in a pouch cell. All spectra are collected in fluorescence mode. The reference powders are applied to Scotch® Tape, which is then folded over. Reference powders do not have pure oxidation states (e.g. the "NiO_2" is actually a mixture of NiO_2 and Ni_2O_3 and therefore has an average XANES of $Ni^{3.5+}$) so that they cannot be taken as absolute benchmarks for the oxidation state of nickel in NCA. However, we observe the correct trends. The lithiated NCA (discharged) state shows XANES similar to NiO and $LiNiO_2$, while delithiated NCA (charged) has an edge higher than that of the NiO_2, indicative of an oxidation state closer to 4+.

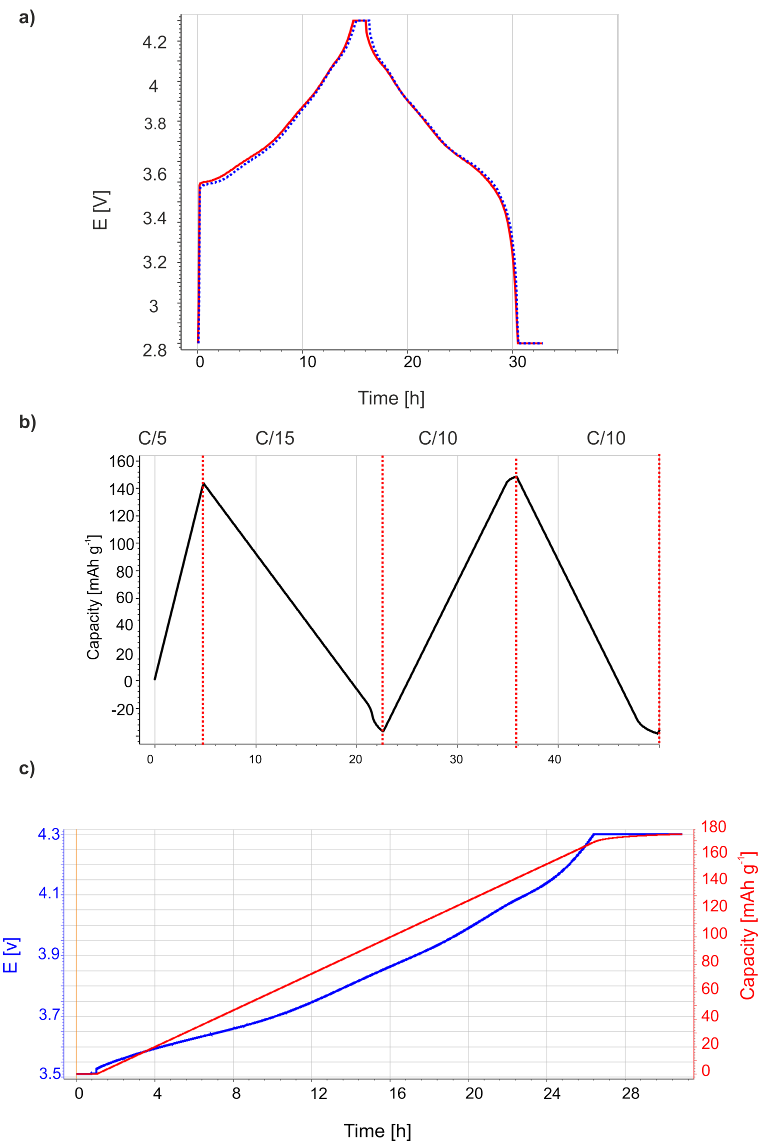

Figure S6 Electrochemistry of pouch cells during the experiment a) Electrochemistry in pouch cell during *operando* XAS measurement (dotted line) is comparable to that of the electrochemistry in a standard laboratory cell (solid line). This is further confirmation that no degradation takes place during the measurement due to the beam. b) Capacity of the cell for the electrochemical cycling presented in Figure 4. c) Electrochemical data of slow charging of a precycled cell from 3.5V to 4.3V in approximately 24 hours for reference measurements.

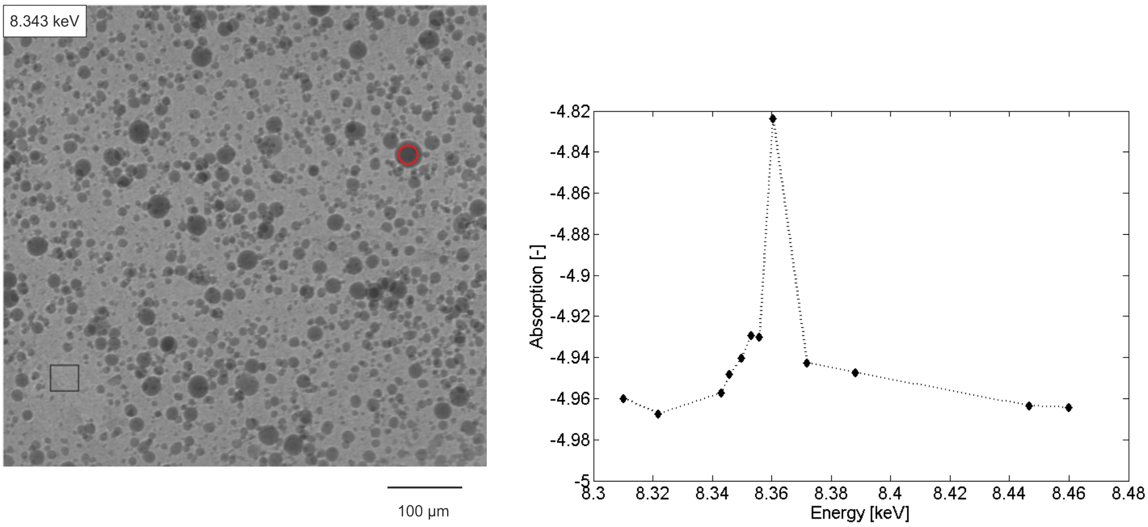

Figure S7 Background normalization of pouch cell attenuation. Black square identifies a background area. The average background attenuation (example right) from the pouch cell (e.g. aluminium layers) is averaged in the square and subtracted from the absorption spectrum.

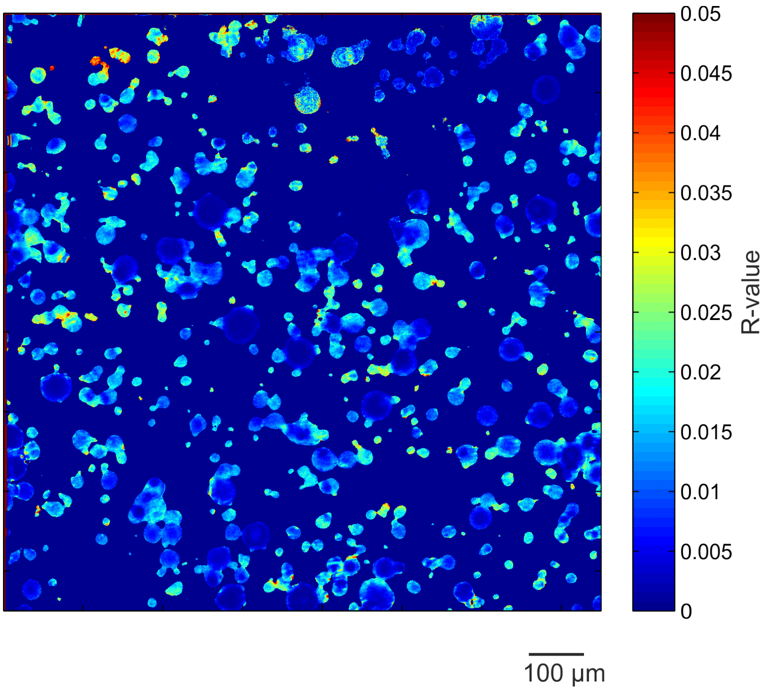

Figure S8 Plot of R-values from attributing pixels to a specific SOC. The average R is lower than 0.02 for all results. The R-values are lower for large particles since there is more material and therefore less noise.

Supplementary Video Transmission images showing the gas bubble that formed during overcharge (with the sample out of the beam) move into the field of view. The "jumps" in the animation correspond to the time where the cell is moved out of the beam for taking background flats.
